# Nutrient Timing: A Garage Door of Opportunity?

**DOI:** 10.3390/nu12071948

**Published:** 2020-06-30

**Authors:** Shawn M. Arent, Harry P. Cintineo, Bridget A. McFadden, Alexa J. Chandler, Michelle A. Arent

**Affiliations:** 1 Department of Exercise Science, University of South Carolina, Columbia, SC 29208, USA; cintineo@email.sc.edu (H.P.C.); bm39@mailbox.sc.edu (B.A.M.); alexajc@email.sc.edu (A.J.C.); 2 Department of Health Promotion, Education, and Behavior, University of South Carolina, Columbia, SC 29208, USA; mgalardi@mailbox.sc.edu

**Keywords:** exercise, sports, performance, recovery, adaptation, nutrition

## Abstract

Nutrient timing involves manipulation of nutrient consumption at specific times in and around exercise bouts in an effort to improve performance, recovery, and adaptation. Its historical perspective centered on ingestion during exercise and grew to include pre- and post-training periods. As research continued, translational focus remained primarily on the impact and outcomes related to nutrient consumption during one specific time period to the exclusion of all others. Additionally, there seemed to be increasing emphasis on outcomes related to hypertrophy and strength at the expense of other potentially more impactful performance measures. As consumption of nutrients does not occur at only one time point in the day, the effect and impact of energy and macronutrient availability becomes an important consideration in determining timing of additional nutrients in and around training and competition. This further complicates the confining of the definition of “nutrient timing” to one very specific moment in time at the exclusion of all other time points. As such, this review suggests a new perspective built on evidence of the interconnectedness of nutrient impact and provides a pragmatic approach to help frame nutrient timing more inclusively. Using this approach, it is argued that the concept of nutrient timing is constrained by reliance on interpretation of an “anabolic window” and may be better viewed as a “garage door of opportunity” to positively impact performance, recovery, and athlete availability.

## 1. Introduction

Nutrient timing is a dietary strategy in which specific nutrients are ingested at certain times surrounding training in order to bolster acute performance and/or chronic adaptations [1]. Much of the early research on this topic assessed the role of acute carbohydrate (CHO) ingestion on exercise performance and rates of glycogen resynthesis to offset glycogen utilization and depletion that occurs particularly during moderate- and high-intensity aerobic exercise [2,3,4,5,6]. Following this work, researchers began investigating the role of acute protein (PRO) ingestion on performance, recovery, and adaptation following both endurance and resistance exercise. However, very few chronic interventional studies have assessed the role of nutrient timing on outcome variables related to performance, recovery, and adaptation. Instead, these early studies coupled with established acute bioenergetic, biochemical, and endocrine responses to exercise were used by Ivy and Portman [7] to provide a theoretical rationale for the role of nutrient timing in chronic adaptations to training.

During a bout of exercise, hormonal fluctuations occur in an intensity-dependent manner to induce various physiological (i.e., increase heart rate and contractility, increase blood flow to working muscles, etc.) and metabolic (i.e., activation of glycolytic and lipolytic enzymes) responses [8]. Hormones including insulin and its counter-regulatory hormones (i.e., glucagon, epinephrine, norepinephrine, cortisol, growth hormone, etc.) exhibit perturbations during exercise. In general, insulin shows a marked decrease, while the secretion of counter-regulatory hormones increases. These changes are metabolically critical for the maintenance of glucose homeostasis and for activating enzymes responsible for increasing mobilization and oxidation of fuels stored within the muscle (i.e., glycogen and intramuscular triglycerides) and throughout the body (i.e., liver glycogen and adipocyte-stored triglycerides). Additionally, independent of insulin, skeletal muscle contraction and the presence of metabolic byproducts stimulate the translocation of GLUT4 to the sarcolemma to further aid in glucose uptake in the muscle [9]. These actions also occur during long-duration, low-to-moderate-intensity exercise. Based on these physiological responses, Ivy and Portman [7] postulated that post-exercise feeding could be used to attenuate the large catabolic shift that occurs during exercise to favor a more anabolic state.

This post-exercise time period was seen to represent an opportunity to enhance adaptation and recovery though nutrition and became known as the “metabolic or anabolic window” due to the enhanced sensitivity of skeletal muscle to nutrient uptake and metabolism. This period was hypothesized to last approximately 45 min while GLUT4 activity remains elevated and glycogen synthase action increases even without stimulation by insulin [10]. This would provide for upregulation of glycogen replenishment. Additionally, in the absence of CHO availability during this period, the proteolytic action of cortisol results in the breakdown of muscle protein and release of alanine to serve as a substrate for gluconeogenesis and, ultimately, glycogen resynthesis [11]. Additionally, the role of protein intake during this period has been emphasized particularly in the context of resistance exercise as mechanical stress coupled with dietary protein or essential amino acids (EAA) alone, especially leucine, lead to significant increases in muscle protein synthesis (MPS) [12].

One of the most prominent nutrient timing studies on the role of muscular strength and hypertrophy was conducted by Cribb and Hayes [13]. They showed that a combined CHO and PRO supplement administered in a roughly 1:1 ratio (e.g., 32 g PRO and 34 g CHO for an 80 kg individual) plus creatine monohydrate consumed both pre- and post-exercise resulted in greater strength and hypertrophy adaptations compared with consuming the same supplement in the morning and at night for 10 weeks in resistance-trained men. Future research used a similar design with regard to timing and outcome measures but did not include CHO in the supplement (42 g PRO only) [14]. No significant differences in performance or body composition were observed between groups. The dichotomous findings between these studies led researchers to believe post-exercise CHO may play a role in chronic strength and hypertrophy adaptations even though acute MPS does not appear to be potentiated with CHO inclusion [15]. While acute metabolic responses were not measured in these studies, the combined CHO and PRO supplement may have had a larger insulinogenic response and, although this hormone does not affect MPS, insulin has been shown to attenuate muscle protein breakdown in the post-exercise period [16]. This anticatabolic effect of insulin may have contributed to the significant differences in body composition changes between groups over the 10-week period in one study but not the other.

The existence of a post-exercise “anabolic window” has recently been called into question [17,18]. A 2013 meta-analysis assessing the role of protein timing on strength and hypertrophy was conducted in an attempt to synthesize the current research [19]. Although the primary finding was that protein timing did not appear to affect strength or hypertrophy outcomes, there are interesting methodological issues to consider. First, of the 23 studies that were included, only 4 studies included resistance-trained subjects. Second, subjects of the included studies came from a large variety of populations, including young men and women, elderly men and women, and older men and women with type 2 diabetes mellitus. Third, many of the studies included did not necessarily assess the role of protein timing but rather tested protein or amino acids alone versus placebo in the peri-exercise period. Lastly, as the authors pointed out, only 2 of the studies included in the analysis equated for total daily protein intake across both treatment and control groups. Conclusions regarding timing impacts would be tenuous at best in this case, particularly since the studies themselves were largely not timing studies. Furthermore, these results have been used to argue that nutrient timing is not important, when, in fact, only one nutrient (PRO) was included and other critical aspects of performance and recovery were not examined. Perhaps more importantly, this study points out the dearth of true nutrient timing studies that exist in trained athletes as well as the need to consider effects that go beyond just hypertrophy and strength.

While the concept of nutrient timing has previously been reviewed [1,20,21,22,23,24,25,26], this has often been done by focusing on singular time periods within the paradigm or organized based on macronutrient impacts rather than holistically as timing. As timing does not occur in isolation, coupled with the fact that the pre-, intra-, and post-training periods interact and influence each other, it is important to consider the concept of nutrient timing across a continuum. Additionally, much of the previous emphasis has been on strength and hypertrophy, despite the fact that athletic performance encompasses so much more than that. The purpose of this review is to present theoretical rationale, current evidence, and practical considerations for various nutrient timing strategies on performance and recovery in healthy, active adults, with a focus on trained athletes. This may help provide a new perspective on a pragmatic approach to maximize outcomes through nutritional intervention.

## 2. Pre-Exercise Nutrition

The pre-exercise nutritional window is often considered to be within approximately <1 h of a training session, though studies have assessed the role of feeding up to 4 h prior to exercise [27]. The primary role of nutrient intake during this time is to ensure adequate fuel is available to the muscle during exercise to optimize performance. Within skeletal muscle, macronutrients, particularly fatty acids and CHO, are required for producing adenosine triphosphate (ATP) to fuel the action of muscular contraction. Fatty acids are the primary fuel source at relatively low exercise intensities (< 60% VO_2max_) as the rate of energy utilization is not particularly high and the speed of ATP synthesis does not need to be extremely rapid [28]. The process of breaking down stored triacylglycerol molecules within adipose tissue (lipolysis via hormone-sensitive lipase), transporting free fatty acids (FFAs) to skeletal muscle mitochondria (intracellular shuttling via carnitine fatty-acyl transferases), oxidizing fatty acid chains into two-carbon molecules (acetyl-CoA), and ultimately, producing ATP via β oxidation and the electron transport chain is a slower process and requires substantial blood flow to adipose tissue, making this possible at low but not high exercise intensities [29]. As exercise intensity increases, blood flow is progressively shunted away from adipose tissue via catecholamine-mediated vasoconstriction, while concomitantly, the rate of ATP utilization increases substantially. Thus, CHO, particularly in the form of muscle glycogen, plays an increasingly important role at higher exercise intensities.

FFA availability is seldom the limiting factor in exercise performance as humans have substantial stores of this substrate in adipose tissue [30]. However, CHO is stored in far lesser quantities throughout the body in the forms of liver (75–100 g or 300–400 kcal) and muscle glycogen (300–500 g or 1200–2000 kcal) and circulating glucose (15–20 g or 60–80 kcal), making glucose availability a limiting factor for work output capabilities [20]. These principles serve as the basis for trying to optimize pre-exercise nutrition through CHO intake. Early research has shown that 2 h of moderate-intensity and 1 h of high-intensity aerobic exercise can deplete muscle glycogen levels by up to 70% [6]. Therefore, it is prudent to increase CHO intake during this period to maximize muscle glycogen levels prior to exercise. This guideline becomes even more important prior to high-intensity or long-duration exercise bouts.

A major concern often associated with pre-exercise CHO feeding is rebound hypoglycemia [31]. This occurs through the combined yet independent effects of insulin, which rises following the consumption of CHO, and muscular contraction on glucose uptake into skeletal muscle via the translocation of GLUT4 to the sarcolemma, resulting in a decrease in circulating glucose. Research, however, shows that rebound hypoglycemia does not occur in all individuals and does not impede acute performance. In fact, the ergogenic effect of consuming additional CHO prior to training or competition appears to outweigh the risks associated with rebound hypoglycemia, and the hypoglycemic effect can be attenuated with a warm-up and small rest period before training or competition [32].

Initial studies on pre-exercise nutrition in sport, dating back to the 1930s, observed physiological responses during exercise following the ingestion of pre-exercise CHO in the form of glucose (GLU) and fructose (FRU) [33]. Soon after, researchers assessed the role of manipulating pre-exercise nutrition on exercise performance. Though Haldi and Wynn [34] showed no differences in performance following various nutritional strategies consisting of supplemental cane sugar in trained swimmers, this study opened the door for many more interventions on this topic. Hargreaves et al. [35] showed no benefit of 75 g CHO (as GLU or FRU only) 45 min pre-exercise on time-to-exhaustion (TTE) performance or glycogen utilization at 75% VO_2max_ in trained cyclists compared with placebo. However, a larger bolus of CHO (312 g) consumed 4 h pre-exercise resulted in an ergogenic effect on time trial (TT) performance at 70% VO_2max_ following 100 min of cycling compared with placebo in recreationally trained cyclists, though the differences were not significant compared with energy-matched meals containing 45 or 156 g CHO [36]. A subsequent study found significant improvements in a similar performance task when recreationally trained subjects consumed 1.1 or 2.2 g/kg CHO 1 h pre-exercise compared with placebo, though no differences were observed between the doses of CHO [37]. Pre-exercise CHO has also been found to affect substrate utilization during exercise. When CHO is consumed, glucose oxidation is favored over FFA even at low-to-moderate intensities (≤ 60% VO_2max_) [38].

Overall, the effects of pre-exercise CHO feeding on endurance performance appear to be beneficial, though the results of some studies are equivocal. However, there are some methodological considerations that need to be considered when interpreting these findings. Major factors which are related and can impact the efficacy of pre-exercise feeding are the time since a previous exhaustive training session and current muscle glycogen content. It can be argued that the importance and ergogenic effects of pre-exercise CHO are highly dependent upon muscle glycogen content prior to that feeding. An individual with limited rest between training sessions and subsequently lower glycogen content likely has much more to gain from a pre-exercise CHO feeding than an individual who has rested for multiple days while consuming adequate CHO during that period. Another factor that may be affecting the efficacy of pre-exercise consumption of CHO is the task that is used to measure performance. For instance, time-to-exhaustion at ≤ 70% VO_2max_ versus a time trial at ≥ 80% VO_2max_ elicits different metabolic responses, with the latter requiring greater oxidation of CHO. Therefore, the efficacy of pre-exercise CHO consumption is likely dependent on the task and intensity of exercise being performed.

Though much of the pre-exercise CHO feeding work has been done using aerobic modalities, it would also seem logical that high-intensity intermittent activities, such as resistance exercise, would benefit as well given their reliance on glycolytic, fast-twitch muscle fibers. These muscle fibers produce high levels of force through rapid muscular contraction, which is fueled by stored phosphagens (ATP and creatine phosphate), and anaerobic glycolysis, which produces lactate. However, total glycogen depletion of only about 40% has been documented following high-volume resistance exercise [39], suggesting that CHO availability is likely not a limiting factor unless glycogen stores are suboptimal to begin with. There is ample evidence demonstrating that pre-exercise CHO supplementation may attenuate reductions in glycogen even if it does not affect blood glucose [40]. Similar to the role of pre-exercise glycogen content mentioned previously, this suggests that performance may be improved by pre-exercise CHO feeding in those with low muscle glycogen content or who may train multiple times per day.

Another important factor to be considered is the modulation of post-exercise metabolic responses by pre-exercise feeding. Although muscle glycogen content does not appear to affect mammalian target of rapamycin (mTOR) pathway signaling and MPS [41], protein (PRO) consumption, particularly in the form of whey, during the pre-exercise period may bolster this post-exercise response [42]. This finding was not observed when only essential amino acids (EAA; 0.35 g/kg FFM) in conjunction with CHO (0.5 g/kg FFM) were provided compared to placebo over a 2-h period [43]. However, when assessing the effects of timing, an EAA-CHO solution (6 g EAA, 35 g sucrose) consumed pre-exercise increased MPS to a greater extent than the same supplement consumed post-exercise [44]. Additionally, Dalbo et al. [45] showed that neither CHO nor PRO consumed pre-exercise augmented ubiquitin-proteasome pathway signaling, an indicator of muscle protein breakdown, in the post-exercise period. In a chronic study assessing the role of PRO timing in resistance-trained individuals over 10 weeks, pre- and post-exercise feedings elicited similar strength and hypertrophy changes when total PRO intake was matched between groups [46]. What has yet to be established is whether feedings in *both* timeframes would be more beneficial. To this point, much of the research has focused on an “either/or” approach to pre-exercise vs post-exercise nutrition.

Aside from performance, an often-overlooked benefit of pre-exercise nutrient intake is the impact on immune function in the context of both endurance and resistance exercise. Chen et al. [47] showed that high CHO pre-exercise meals (104 g CHO) resulted in less disruption in immune cell counts and IL-6 in the 2–h post-exercise period compared to low CHO (56 g CHO). Differential effects on markers of immune function of supplemental CHO in not only the pre-exercise period but also intra- and post-exercise have been found [48]. However, it appears as though CHO offers more favorable impacts on salivary immunoglobulin A during high-repetition, high-volume resistance exercise compared to moderate-volume bouts. This is likely due to the immunosuppressive effects of hormonal responses that occur during high-intensity resistance exercise but do not occur at low intensities [49]. The beneficial effects on performance may be indirect in this case, as a healthier athlete may be more available for training and competition. Given the current global status and the SARS-CoV-2 pandemic, this is an effect that should not be overlooked but that has largely not been considered in previous reviews on nutrient timing.

The importance of pre-exercise nutrition is contingent on glycogen and energy status as well as the time since previous feeding. The timing of this pre-exercise meal becomes incredibly important for an individual who is glycogen-depleted and will be undergoing exercise that would typically require a high degree of glycogen contribution. Additionally, pre-training consumption of both CHO (0.5–2.2 g/kg) and PRO (0.3–0.35 g/kg) can aid not only in maximizing acute performance, but also in facilitating recovery and adaptation from that training. This likely depends on the quantity and timing of post-exercise protein intake as well.

## 3. Intra-Exercise Nutrition

The provision of CHO during activity is likely the most well-studied nutrient timing strategy, dating back to the 1960s [50]. CHO intake during the exercise can offset muscle and liver glycogen utilization and maintain blood glucose. This is especially important when exercise intensity is high, the duration exceeds 60 min, or during shorter, supramaximal efforts [51,52]. In these scenarios, without adequate CHO, exercise intensity will diminish [2] potentially due to lack of effective fuel, reduced calcium release from the sarcoplasmic reticulum, and fatigue [53].

Exogenous CHO oxidation rates increase exponentially during the first 75–90 min of exercise, indicating that CHO ingestion from the onset and throughout the exercise bout may aid in the sparing of muscle and liver glycogen [54]. However, large CHO intake in these conditions can create GI upset, which would be counterproductive to performance goals. It has been well established that maximum rates of oxidation of glucose (GLU) alone are 1 g/min, leading to roughly 60 g/h [54]. However, ingestion of multiple types of CHO makes use of different transporters and results in an increased capability of CHO uptake and, thus, oxidation to roughly 1.5 g/min or 90 g/h [55]. Consumption of multiple transportable CHO not only results in increased CHO availability without GI upset [56] but may also improve performance [57]. In fact, an 8% higher power output was achieved during time trial following 120 minutes of steady state cycling when cyclists consumed 2:1 GLU:FRU beverages compared to consuming GLU alone [57]. It should be noted that FRU ingested at 1.2 g/min combined with an equal quantity of GLU results in a higher CHO oxidation rate compared to ingestion of lesser quantities that would still meet the common 60 g/h recommendations. This highlights the potential utility of higher intake rates of FRU and GLU to maximize CHO oxidation [55,58,59]. It is possible that recommendations for CHO consumption during exercise should be revisited based on these findings, as it appears higher rates, such as 90–144 g/h during long-duration events may be needed to maximize uptake via the unique glucose and fructose transporters [55,58,59,60]. Furthermore, ingestion of 120 g/h of a 2:1 GLU:FRU solution during a mountain marathon was found to decrease markers of muscle damage as well as rating of perceived exertion (RPE) compared to consuming either 60 or 90 g/h [61], suggesting the positive effects of higher intakes go beyond just performance.

As our understanding of mechanisms underlying glycogen utilization and replenishment has grown, so too has the consideration of manipulation of CHO sources, ratios, and delivery methods in order to maximize the body’s ability to absorb and utilize the substrates for fuel as a means to enhance acute and chronic performance over time [54]. A new CHO supplement composed of maltodextrin and FRU (1:0.8) in an alginate and pectin gel debuted to the general public during the chronicling of the efforts to break the 2–h marathon and had the sport supplement world buzzing with prospects of increasing CHO uptake and utilization. Proposal of the use of alginate, a derivative of seaweed, in an effort to encapsulate CHO to allow passage from the stomach to the small intestine without causing GI distress led to a proof-of-concept pilot study conducted in 16 well-trained Kenyan runners [62]. Four of the 16 runners were provided with 180 g/L or 300 g/L CHO alginate gel while all athletes were provided with 40–50 g/L CHO every 5 km, and supplements were consumed ad libitum. No reports of performance differences between athletes were indicated, however preliminary data from the pilot suggest that no GI issues resulted from ingestion of alginate-based CHO. While measurement of CHO oxidation rates did not occur during the attempt to break the 2–h marathon or the aforementioned pilot study, such research utilizing the unique CHO hydrogel with a concentration of 18% was conducted in elite cross-country skiers who performed a 2–h exercise bout at 70% VO_2peak_ followed by a TT [63]. Results shows that compared to placebo, consumption of the hydrogel resulted in increased rates of exogenous CHO oxidation, decreased fat oxidation, and decreased usage of endogenous CHO. Despite these findings, no performance differences were seen during TT, potentially suggesting that CHO oxidation may have an “upper limit” in terms of performance impacts. However, speculation regarding the effectiveness of the glycogen depleting protocol has been noted, indicating additional research is required to determine the important of consumption of an 18% CHO hydrogel on performance.

Another strategy to deliver CHO while minimizing GI distress and possibly further improve exercise performance is to co-ingest PRO and CHO. A recent review and meta-analysis by Nielsen et al. [64] demonstrated favorable outcomes on performance during time trials or time to exhaustion efforts in groups consuming combined CHO-PRO versus CHO alone. This effect remained when non-isocaloric supplements (CHO-PRO vs CHO alone) were consumed as well as when CHO-PRO and CHO supplements were matched for CHO content. However, when investigating the effects of isocaloric supplementation of CHO-PRO or CHO alone on time to exhaustion, no differences were seen [64]. While co-ingestion of PRO and CHO may not result in direct, acute performance improvements, indirect benefits include the ability to increase caloric consumption while decreasing CHO intake to avoid GI distress, the increase of amino acid (AA) bioavailability to decrease rates of muscle protein breakdown, the increased AA availability and utilization for gluconeogenesis, and even delay of central nervous system (CNS) fatigue [65]. The Central Fatigue Hypothesis suggests that as skeletal muscle selectively oxidizes branched chain amino acids (BCAAs) during prolonged exercise, free tryptophan crosses the blood-brain barrier increasing serotonin concentrations in the brain, which may lead to impaired exercise performance via central fatigue [66]. Consuming PRO during exercise alters the free tryptophan to BCAA ratio, resulting in decreased serotonin and delayed onset of central fatigue [66]. This may in part explain the findings of the meta-analysis by Stearns et al. [67], who concluded that PRO and CHO improved TTE but not TT performance. The authors speculate that TTE may rely on more psychological factors than a TT, such as boredom and lack of motivation, leading to the suggestion that there may be a CNS effect of PRO on endurance performance [67].

Preventing central fatigue and cognitive decline may be especially important during activities such as sporting matches, where decreases in blood glucose are related to impaired cognitive function [68,69]. This effect may be compounded by the fact that opportunities to consume CHO may be limited during these performances, further increasing the importance of maximizing initial glycogen stores and utilizing effective feeding strategies when possible during the match. Seminal work by Saltin [69] demonstrated intermittent, high-intensity activity lasting > 60 min results in diminished glycogen stores. When compared to pre-match levels, muscle glycogen was reduced by 67% at half-time and by > 90% upon the conclusion of a soccer match [69]. Glycogen depletion in these athletes is related to decreased concentration and mental acuity as well as increased fatigue [68,69]. Research demonstrates positive effects on cognition and improved performance under fatiguing conditions during prolonged sport matches as a result of pre-game and half-time feeding strategies [68,70]. Additionally, Russell et al. [71] found that CHO feeding during a simulated soccer match attenuated decrements in shooting performance and positively impacted blood glucose. Kingsley et al. [72] found that adding caffeine to a CHO/electrolyte gel improved sprint speed during a simulated soccer match compared to placebo, but it had less favorable impacts on hydration status than the CHO/electrolyte gel without the added caffeine. As with previous work, blood glucose was also favorably impacted. Notably, the sparing of liver glycogen plays an essential role in maintaining blood glucose levels and hepatic glucose output [11], ultimately ensuring adequate CHO is available for the brain to fuel cognitive processing [73]. Another often overlooked, but particularly important, benefit of CHO consumption during training is that it may also aid in reduction of immune suppression that can occur [74].

There is a persistent misconception that long-duration endurance events are primarily fueled by fat oxidation and, as such, would be better served by feedings or adaptions that would favor this rather than CHO feeding and oxidation. While investigations into ketone-fueled endurance events show promise that completion of such events is possible, direct comparison of CHO- versus ketone-fueled race-walking performances demonstrate advantage to those who are fueled by CHO [74,75]. Support of “keto-adaptation” for athletic performance is often attributed to higher rates of fatty acid oxidation that occur in this state. However, the more pragmatic question to ask is if increased fat oxidation results in improved performance. Results from the aforementioned studies would suggest not. In fact, the increased fat oxidation that occurred during the exercise trial resulted in decreased exercise economy and a greater cost of performance to the athlete. Further, over the course of the 3-week intensified training protocol, the high-fat, low-CHO group was the only group that did not improve performance while the high- and periodized-CHO groups both demonstrated improved performance. For this strategy to yield benefit, exercise intensity must be appropriate for fat oxidation to occur, but contrary to popular thought, this is not always the case. Additionally, when in a “keto-adapted” state, the beneficial effects of supplemental CHO during exercise discussed throughout this section are attenuated as low-CHO, high-fat diets decrease activity of the pyruvate dehydrogenase complex, the enzyme complex required to convert pyruvate to acetyl-CoA and initiate the complete aerobic oxidation of GLU [75].

Although not as substantial as endurance exercise lasting > 60 min, resistance exercise can result in muscle glycogen depletion by 17–40% depending on the duration and intensity of the work bout [76,77]. Reduced muscular power and strength resulting from depleted muscle glycogen stores have led to recommendations for CHO consumption during resistance training as a means to increase blood glucose concentrations and reduce muscle glycogen depletion [39]. Overall, evidence suggests the largest ergogenic benefits of CHO consumption during resistance training will occur during high intensity exercise characterized by achieving repeated muscular failure and bouts where duration is > 40 min as these may have greater impacts on muscle glycogen depletion [39]. While consumption of CHO during a resistance training session has been shown to prevent muscle glycogen depletion, this may not translate to any improvement in performance metrics within the same training session [76]. However, consumption of CHO (3 g/kg) during a morning resistance training session has been shown to result in improved performance on number of sets-to-exhaustion for squat in a subsequent training session 4 h later [39]. While the acute performance benefits of CHO supplementation during resistance exercise may be limited to longer duration, high intensity work bouts, the positive impacts on subsequent training sessions cannot be ignored. This strategy may be of particular importance for athletes training multiple times per day. Additional benefits during the early recovery period may been seen as a result of CHO and PRO intake during training [39]. Research suggests that consuming PRO or branched-chain AAs (BCAAs) with CHO before or during resistance training can lead to greater MPS increases during the early post-exercise period compared to post-exercise supplementation [78]. Similarly, intra-workout CHO or EAA feedings have been found to positively impact hormonal responses and muscle protein degradation, but a combination of the two produced the greatest acute and chronic effects [79,80].

Few studies have assessed application of within-session timing of CHO consumption., McConell, Kloot, and Hargreaves [81] found that when trained cyclists consumed 157.5 g CHO over the course of an entire exercise bout, as opposed to only within the final 30 min, they improved 15-min TT performance following a 2–h cycling bout at 70% VO_2peak_. As this study used a dosage 78.25 g/h of a 21% CHO beverage solution, which is larger than the current recommendations (30–60 g/h of 6–8% CHO solution)its results inspired a follow-up study employing a similar design but with 75 g (50 g/h) of a 6% CHO beverage [82]. This follow-up study found improved TT performance following a 2–h cycling bout when 75 g CHO was ingested using a front-loading (every 15 min during first hour) protocol compared to a continuous loading (every 15 min throughout the bout), or back-loading (every 15 min during second hour) protocol [81]. It may be hypothesized that differences are due in part to spared endogenous CHO during the front-loading protocol, while consumption of 75 g after partial glycogen depletion may not be sufficient to offset additional glycogen depletion or drop in blood glucose. Consumption of 75 g CHO over the course of 2 h is on the lowest end of the current recommendations and is likely inadequate to offer benefit. Together, these data lend support to the need to revisit within-exercise CHO consumption recommendations.

The efficacy of intra-exercise nutrition, particularly CHO, is highly dependent on pre-exercise feeding, glycogen status, and the type of exercise. For aerobic exercise lasting ≥ 2 h, consumption of 90–144 g/h CHO in the form of a 2:1 GLU:FRU solution appears to maximize CHO uptake and oxidation while also sparing muscle glycogen. This becomes extremely important during competition, as long endurance bouts typically conclude with a sprint to the finish line. This relies heavily on anaerobic metabolism and the oxidation of endogenous muscle glycogen. Thus, sparing this fuel source throughout the bout becomes critical.

## 4. Post-Exercise Nutrition

The post-exercise period is often associated with temporary increases in fatigue and muscle soreness, and decrements in performance. During this time, catabolic processes predominate resulting in elevated cortisol and catecholamines, low insulin, reduced glycogen and substrate availability, and increased rates of muscle protein breakdown [20]. Post-exercise CHO and PRO intake have the ability to increase blood glucose levels, decrease cortisol, and increase substrate availability, thus amplifying the body’s shift from a catabolic to a more anabolic state [20]. In addition, the activation of muscle GLUT4 transporters, increased glycogen synthase activity, and enhanced insulin sensitivity increase the responsiveness of skeletal muscle to CHO and AA uptake [83,84]. Therefore, the period following exercise provides an ideal opportunity for timed nutrient intake in order to promote the restoration of muscle glycogen and protein synthesis, while helping to reduce muscle protein breakdown [20,83,85]. In doing so, post-exercise nutrient timing may be an essential aspect of an optimal training program as it has the potential to improve the rate of recovery and maximizes training adaptations.

During exercise of moderate-to-high intensity, muscle glycogen stores represent the most important fuel source to sustain exercise. In these scenarios, post-exercise nutrient timing should largely focus on the restoration of muscle glycogen to improve rates of recovery. When exercise stops, the increase in post-exercise glucose transporters begins to decline and returns to baseline levels within 2 h [86]. The upregulation of GLUT4 transporters immediately following exercise provides a window of opportunity to take advantage of the muscle’s ability to uptake glucose and optimally replenish glycogen stores. It has been suggested that an optimal restoration of muscle glycogen post-exercise can occur through CHO intakes of 1.0–1.5 g/kg/h [83,87,88] initiated within the first 2 h after the cessation of exercise [3], and should continue for 4 to 6 hours with more frequent feedings (15–30 min intervals) being favorable for maximal glycogen resynthesis [83,84,89]. In addition, high glycemic index (GIx) CHO may be optimal for rapid muscle glycogen resynthesis [90,91] as they have been shown to produce a higher insulinemic response than low GIx CHO [92]; however, mixed results have been found regarding the effects of high GIx CHO on subsequent performance bouts [92,93]. It important to note that nutrient timing post-exercise is highlighted by the need to restore glycogen levels and becomes increasingly important when rapid restoration of glycogen is required, as such with multiple bout competitions or when insufficient CHO are being delivered to meet the daily energy goals [94]. It may not be as essential when CHO intake is sufficient to match energy demands [94]. However, timed ingestion of post-exercise CHO has never been shown to have negative implications on performance. It may also be crucial for athletes with a demanding training schedule over the course of the week, as well as for those training multiple times per day.

In addition to the beneficial effects of CHO to optimize glycogen stores, CHO intake accompanying exercise has been shown to attenuate various markers of muscle breakdown and cytokine production, thus improving inflammatory recovery [48,95,96]. The immunoprotective properties of CHO may be important during times of high-intensity or long-duration exercise which has been shown to suppress the immune system [49,95,96], leaving an athlete susceptible to illness. Rapid ingestion of CHO post-exercise may aid in restoring the immune system, particularly after high-intensity or strenuous exercise. As previously noted, this has important ramifications for athlete availability for training and competition and has particular relevance in the current climate of the SARS-CoV-2 pandemic.

While CHO intake post-exercise plays a key role in glycogen resynthesis and immune protection, PRO is another macronutrient essential to post-exercise recovery. The co-ingestion of PRO and CHO has been shown to further increase insulin secretion leading to increased muscle glycogen synthesis [83,89,97]. The addition of PRO (0.4 g/kg/h) to CHO may stimulate glycogen synthesis to a greater extent than CHO alone, specifically if CHO intake is < 1.0–1.2 g/kg/h [83,89,98]. PRO (0.4 g/kg/h) combined with CHO (0.8 g/kg/h) within 2 h following exhaustive exercise has also been shown to improve subsequent cycling performance compared to CHO intake alone in endurance-trained men, suggesting improved recovery and restoration of fuel stores [99]. This may become particularly relevant in situations in which it may be difficult to consume optimal CHO amounts between multiple exercise bouts due to time constraints or GI problems, which may be a common concern with high CHO intakes [100]. Additional consideration for other forms of supplementation may also be warranted to improve glycogen resynthesis. For example, adding 2 mg/kg/h caffeine to 1.0 g/kg/h CHO intake over a 4–h post-exercise period has further shown to increase glycogen synthesis rate by 66% compared to CHO alone [101], with additional improvements in subsequent high-intensity interval running capacity shown in recreationally active men ingesting 1.2 g/kg CHO with 8 mg/kg caffeine [102].

In addition to glycogen synthesis, PRO and EAA following exercise play a critical role in stimulating MPS and allow for skeletal muscle reconditioning [103]. The post-exercise period is characterized by increased muscle damage and protein breakdown [20,83]. Further, glycogen depletion increases the rate of protein degradation as AAs may undergo gluconeogenesis and be used to restore glycogen levels [104]. Therefore, protein intake post-exercise is critical to reduce protein breakdown and help to repair muscle damage [103]. With regard to stimulating MPS, rapidly-digestible, high-quality proteins containing sufficient EAAs may be more efficient compared to lower-quantity branched chain amino acids (BCAA) or slower digested proteins [105]. The exact amount of PRO required for optimal MPS post-exercise is unclear as this may depend on the athlete as well as the exercise session. PRO doses of 20 g from a high-quality, fast-absorbing source have been shown to maximize MPS following resistance exercise [106,107] and high-intensity aerobic exercise [108]. However, a follow-up study by MacNaughton and colleagues [109] compared 20 versus 40 g of whey PRO following whole-body resistance exercise in resistance trained young men and found 40 g stimulated MPS to a greater extent, highlighting the influence of the training session itself and the need for amino acid delivery. It is important to note that CHO intakes post-exercise have been shown to attenuate muscle protein breakdown but have not been shown to affect MPS [83,110,111].

Although post-exercise PRO plus leucine supplementation has been shown to saturate BCAA metabolism and decrease tissue damage, the effects on subsequent intense endurance performance were found to be trivial leading to the conclusion that post-exercise supplementation may be inconsequential when daily PRO consumption is sufficient to induce positive nitrogen balance [112]. It has also been suggested that the timing of protein intake post-exercise may not be important to maximizing MPS, as total PRO intake is more important than post-workout timing *per se* for strength and hypertrophy [17]. The fact that exercise-induced MPS is elevated for 24–48 h following high-intensity aerobic [113] and resistance exercise [114] has been used to bolster this position and argue that an “anabolic window” does not truly exist [19]. However, this fails to acknowledge that this larger window actually represents an *opportunity* to enhance the effectiveness of multiple feedings. Given the beneficial effects of protein to restore net protein balance post-exercise as well as the beneficial effects on glycogen synthesis, it appears there are advantages to PRO timing immediately post-exercise. The bottom line is that quality of training and total PRO intake in a day are more important than acute post-workout protein ingestion for strength and hypertrophy, but this becomes more of a hierarchy issue. Once training quality and total PRO intake are both accounted for, PRO timing may provide the added support to optimize performance. Even if it provides only small benefits, this may be an important training consideration for competitive athletes looking to optimize performance. In novice training populations which tend to be overrepresented in this literature, the effects may be far less impactful.

Ultimately, optimal post-exercise nutrition will largely depend on the type of exercise and the intensity, duration, and frequency of the exercise bouts. The magnitude of the training stimulus and resultant glycogen depletion and protein breakdown becomes particularly important for athletes who are required to perform multiple sessions per day. Therefore, feeding between sessions becomes increasingly important to ensure sufficient fuel stores for the subsequent exercise bout and overall recovery and adaptation. Both the intensity and duration of training will also influence the importance of timing as well as the nutrients needed for optimal responses. For those undergoing glycogen-depleting exercise, 1.0–1.5 g/kg/h CHO for 4—6 h following exercise or the combination of 0.4 g/kg PRO when CHO intake is < 1.0 g/kg/h appears to maximize glycogen replenishment, attenuate MPB, and optimize recovery. Following resistance exercise, ≥ 40 g PRO has been suggested to maximize MPS rates and reduce MPB although the exact amount will largely depend the individual as well as the exercise bout. The addition of 0.5 g/kg CHO also appears to be beneficial during this time to further reduce MPB.

## 5. Additional Timing Considerations

Early nutrient timing research on the efficacy of consuming specific nutrients surrounding an exercise bout has emphasized the importance of the peri-exercise time period. However, the remaining hours of the day comprise of the majority of the day, and nutrient intake during this time cannot be ignored. Fueling consistently throughout the day has been shown to be an effective strategy for maximizing performance at later times. For instance, although strategies such as skipping breakfast or time-restricted feeding may be effective for inducing weight loss [115,116], training in a fasted state results in worsened performance, particularly during prolonged exercise, compared to training in a fed state [117,118]. Further, omitting breakfast has been shown to attenuate performance in recreationally active adults even after lunch has been consumed [119].

Within the context of protein intake, performance, and body composition, the idea of protein pacing has been researched extensively in both overweight and obese individuals [120,121,122] as well as healthy, fit individuals [123]. Protein pacing refers to the concepts of continuously and consistently feeding PRO throughout the day in order to maintain maximal MPS rates and optimize recovery, adaptation, and performance. Though few data exist on the application of this strategy in athletes, this strategy is supported by detailed studies in healthy adults investigating protein consumption throughout the day in a balanced manner (spread evenly across meals at 30 g/meal) or in a skewed manner (10 g at breakfast, 15 g at lunch, 65 g at dinner) revealed increased protein synthesis in the groups consuming evenly distributed protein [124]. Additionally. work done by Arciero and colleagues [125] demonstrated that protein consumption evenly distributed in 6 meals throughout the day had significantly greater impact on metabolic markers than did the consumption of the same amount of protein consumed in 3 meals thought the day. It should be highlighted that the improvement in cardiometabolic markers were accompanied by increases in lean mass and drops in body fat and abdominal fat, while bodyweight remained unchanged [125]. In essence, the positive impact of evenly spaced protein consumption throughout the day was independent of weight loss.

Additionally, research on the ingestion of protein prior to sleep has suggested that this strategy is effective in maximizing overnight MPS rates, facilitating recovery and adaptation during this period [126,127]. Nighttime feeding of PRO and CHO in the form of chocolate milk has also been shown to affect morning metabolism, though subsequent day time trial time was not improved compared to placebo [128]. Comparing the efficacy of morning versus nighttime feeding of 56 g PRO from casein, however, showed no differences in muscular strength or endurance over 8 weeks of resistance training when subjects were consuming PRO in the quantity of 2.6 g/kg/day [129]. In this scenario, it is unlikely that nighttime feeding would have an appreciable effect given the overriding importance of total daily intake. Perhaps in lesser total quantities, this approach may be more impactful. Despite these results, it is clear that timing the consumption of nutrients throughout the day may offer certain benefits, even if not directly impacting performance, above simply focusing on the peri-exercise period. There is even evidence that timing/order of nutrient intake *within a meal* can impact metabolic responses, blood glucose, and insulin [130], which may have implications for nutrient absorption.

Recently, the concept of periodized nutrition has been promoted. Using this model, dietary intake is altered throughout training micro-, meso-, and macrocycles [25]. This model helps bridge the gap between acute performance and chronic adaptations. This may also be a useful approach for athletes needing to lose weight for competition and to promote lean mass gains during certain training phases. This approach can also be extended to include the concept of “CHO-restricted” training. This involves intentionally training in a state of low CHO availability and glycogen levels. Though this may attenuate performance within the acute training bout, it has been shown to potentially bolster long-term adaptations [25,131]. Such adaptations include mitochondrial biogenesis, increased capillarization, and increased capacity for lipid oxidation [25], all of which may contribute to improved endurance performance. In other words, there may be scenarios where “optimal” fueling during training is not always desirable in order to produce “optimal” adaptations. However, a few words of caution are warranted here. First, the studies on this approach have been relatively short in duration and have not applied it as an “all the time” solution to training. It is generally limited to select training sessions per week, often consisting of the steady-state training sessions on a morning following a high-intensity glycogen depleting session [132]. Second, this has almost exclusively been studied in endurance athletes (i.e., cyclists or runners) or using an endurance exercise protocol [132]. The applicability to power-endurance and team-sport athletes should be questioned due to potential concerns over injury when explosive movements or contact are involved. Finally, performance has not consistently been found to be improved [132]. When it has been, the protocol typically involved PRO feeding prior to the CHO-restricted bout [132,133,134], which would still argue for the importance of nutrient timing.

## 6. Practical Implications

What has often been interpreted as lack of support for the importance of nutrient timing has largely been based on studies in novice training populations and “non-timing” studies, or on noted equivocal findings. However, the equivocal nature of these findings is primarily due to the magnitude of an effect, not on complete absence of an effect or, more importantly, negative effects. In trained individuals and athletes, the overriding conclusion that should be drawn from this body of literature is that there never appears to be a disadvantage associated with feeding appropriately at any time surrounding an exercise bout. Additionally, beneficial effects may manifest themselves over time due to recovery considerations. The greatest ability of an athlete is availability. Effects on immune function have received little attention in previous reviews on this topic. We must also be cognizant of the fact that “nutrients” are more than just one single macronutrient. It would be prudent for athletes to attempt to optimize nutrient timing based not only on what has been tested directly but also on our mechanistic understanding of physiological responses to exercise both in an acute and chronic sense. In addition, the peri-exercise period, particularly in athletes who train multiple times per day and have busy schedules outside of training, should be viewed as a window of opportunity to feed to get closer to one’s individual daily energy and macronutrient goals. In many cases, if the peri-workout period is not utilized for feeding, it will be almost impossible for athletes and hard-training individuals to adequately hit their total daily intake needs.

**Figure 1:**
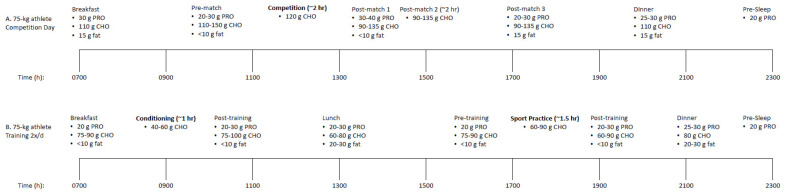
Sample nutrient timing strategy for a 75-kg power-endurance athlete (i.e., soccer) for (A) competition day and (B) conditioning session + team practice.

Further, in the context of sports nutrition for optimizing performance and recovery, the issue of consuming nutrients should not be separated into “before, during, *or*, after” but should be combined as “before, during, *and*, after.” Though research suggests that consuming certain nutrients at certain times may be more beneficial than other times, there is likely the most to gain from consuming nutrients at all times surrounding exercise bouts and throughout the day. As mentioned previously, this concept becomes most important once individuals have determined and consistently consumed adequate total energy and macronutrients throughout the day. Athletes, particularly those who train multiple times per day, participate in glycogen-depleting exercise during training and/or competition, and incur substantial amounts of muscular damage, should look at the peri-exercise period as well as the remaining times throughout the day as opportunities to consume nutrients such as CHO and PRO to help facilitate recovery, adaptation, and ultimately performance. Frequent and consistent CHO and PRO feedings appear to have notable positive benefits [12,123]. Examples of nutrient timing strategies for a power-endurance athlete, such as a soccer player, on match and training days can be found in Figure 1.

Macronutrient sources are also important to consider, particularly in the peri-workout period. Much of the research discussed thus far has provided high-glycemic, low-fiber, fast-absorbing carbohydrates (i.e., dextrose, maltodextrin, etc.) before, during and immediately post-exercise. These CHO sources allow for rapid GI transit time and gastric emptying to allow for rapid release into the blood and uptake by the muscle for oxidation or glycogen synthesis. Additionally, the role of protein supplementation has been recently reviewed [26], and it is clear that high-quality, fast-absorbing whey protein derived from dairy appears to be the most convenient protein source in the post-exercise period to maximally stimulate MPS. Whey protein also appears to stimulate post-exercise MPS to a greater extent than soy protein when consuming a protein-matched (30 g) dose [135], which remains consistent even when EAA content (10 g) is matched [107].

In conclusion, nutrient timing is a nuanced topic, and its importance is highly context dependent. The scenarios in which specific nutrient intake becomes important have been discussed throughout this review. For instance, if one’s glycogen levels are low or depleted prior to training or competition, consuming CHO prior to exercise will have a much greater effect on acute performance than for an individual who has already sufficiently replenished glycogen. Additionally, the consumption of PRO immediately post-exercise is likely much more important for stimulating MPS and attenuating MPB for an individual who resistance-trained in a fasted state versus one who had a pre-exercise meal consisting of adequate PRO and CHO. Ultimately, once individual total daily energy and macronutrient needs are assessed and determined, nutrient timing strategies can be implemented in accordance with the current evidence which shows that feeding consistently throughout the day, particularly in the peri-exercise period, is the most optimal strategy for maximizing performance. It is fair to question the nature of the “anabolic window” based on our current understanding of protein metabolism and the stimulus provided by resistance training. However, if anything, it would simply appear that this window is much longer than originally proposed and may in fact be more like a “garage door”. Unfortunately, this has been used to argue that post-exercise refeeding is not *essential.* However, it may be optimal and represents an *opportunity* to improve adaptation and recovery. When it comes to nutrient intake for athletes and active individuals, there exists a hierarchy of needs. If we look at it like baking a cake, the training stimulus and the total daily intake form the cake itself. The timing of nutrient intake is more like the frosting, which requires the foundation of the cake to do its job. Finally, the more advanced concepts such as nutrient periodization and CHO-restricted training are the decorations on the cake. Most importantly, proper feeding around training, despite questions of magnitude of benefit, is never detrimental.
